# Association between escitalopram dose personalisation based on quantification of drug plasma levels and the outcome of escitalopram treatment

**DOI:** 10.1192/j.eurpsy.2024.145

**Published:** 2024-08-27

**Authors:** P. G. Vuković, A. Jeremić, M. Vezmar, D. Pešić, B. Pejušković, J. Drakulić Đorđević, F. Milosavljević, C. Miljević, N. P. Marić - Bojović, B. Marković, M. Ingelman - Sundberg, M. M. Jukić

**Affiliations:** ^1^Department of Physiology, University of Belgrade - Faculty of Pharmacy; ^2^ Institute of Mental Health; ^3^Department of Psychiatry, University of Belgrade - Faculty of Medicine; ^4^Department of Pharmaceutical Chemistry, University of Belgrade - Faculty of Pharmacy, Belgrade, Serbia; ^5^Pharmacogenetics section - Department of Physiology and Pharmacology, Karolinska Institutet, Solna, Sweden

## Abstract

**Introduction:**

Given the negative impact of anxiety and depression on society and the shortage of new antidepressants, it is of paramount importance to make the best use of available treatment options. Therapeutic drug monitoring (TDM) in escitalopram treatment can potentially be clinically useful, as underexposed patients show reduced efficacy of escitalopram treatment and as adverse drug reactions (ADRs) of escitalopram are dose-dependent.

**Objectives:**

This prospective cohort study aimed to investigate whether escitalopram treatment efficacy or safety are associated with escitalopram dose adjustment based on TDM readouts.

**Methods:**

89 included patients aged between 15 and 65 years who suffered from depression were enrolled in the study before starting treatment with escitalopram. Patients were assessed one day before starting treatment with the recommended dose of 10 mg/day escitalopram (baseline, visit 0) and at follow-up after four and eight weeks. Dose adjustment at four-week follow-up was based on the measured escitalopram plasma level two weeks after treatment initiation; patients who required dose increase to 15 or 20 mg/day comprised comparator group, patients who did not required dose increase comprised control group, while patients who did not reach optimal exposure at eight-week follow-up were characterized as non-compliers. Treatment efficacy was approximated by the relative change on the Hamilton Depression Rating Scale (HAMD), while safety was approximated based on the changes on the Scandinavian UKU side effect rating scale and ECG readouts. Changes in HAMD, UKU score and QTc interval were compared between groups by one-way ANOVA or chi-square tests.

**Results:**

Compared to baseline, significant reductions in HAMD scores of 36% (95%CI, 30%-43%) and 53% (95%CI, 47%-60%) were observed at four- and eight-week follow-up, respectively; however, there were no significant differences between groups (p > 0.1). In the groups adjusted to 15 and 20 mg, 15/26 and 19/33 patients, respectively, reported adverse effects, compared with 6/17 patients in the control group and 6/13 in the non-complier group (*p*>0.1). A significant mean QTc prolongation of 6.40 ms (95%CI, 3.27-9.53) was observed between the baseline and eight-week follow-up (*p*=0.0013), without significant differences in QTc interval prolongation between groups (p > 0.1).

**Conclusions:**

Escitalopram dose adjustment resulted in optimal drug exposure and solid treatment response in the majority of patients; however, no differences in efficacy were found between the patients who required dose adjustments, the ones who did not, and the ones who ultimately did not achieve optimal exposure. In addition, the selective increase of the dose to the patients who did not reach optimal drug exposure on the recommended dose of 10 mg/day did not lead to significant increase in adverse drug reactions and QTc prolongation.

**Disclosure of Interest:**

None Declared

